# Anatomical and molecular insights into the antennal gland of the giant freshwater prawn *Macrobrachium rosenbergii*

**DOI:** 10.1007/s00441-024-03898-3

**Published:** 2024-06-15

**Authors:** Thanapong Kruangkum, Kornchanok Jaiboon, Phakkhananan Pakawanit, Jirawat Saetan, Arnon Pudgerd, Suttipong Wannapaiboon, Charoonroj Chotwiwatthanakun, Scott F. Cummins, Prasert Sobhon, Rapeepun Vanichviriyakit

**Affiliations:** 1 Department of Anatomy, Faculty of Science, Mahidol University, Bangkok, 10400 Thailand; 2 Center of Excellence for Shrimp Molecular Biology and Biotechnology (Centex Shrimp), Faculty of Science, Mahidol University, Rama VI Road, Bangkok, 10400 Thailand; 3 Synchrotron Light Research Institute (Public Organization), Nakhon Ratchasima, 30000 Thailand; 4 Division of Health and Applied Sciences, Faculty of Science, Prince of Songkla University, Hat Yai, Songkhla, 90112 Thailand; 5 Division of Anatomy, School of Medical Science, University of Phayao, Muang, Phayao, 56000 Thailand; 6 Mahidol University, Nakhonsawan Campus, Nakhonsawan, 60130 Thailand; 7 Centre for Bioinnovation, University of the Sunshine Coast, Maroochydore DC, Sippy Downs, QLD 4558 Australia; 8 School of Science, Technology and Engineering, University of the Sunshine Coast, Maroochydore DC, Sippy Downs, QLD 4558 Australia

**Keywords:** Crustacean, Nephopore, Kidney, Bladder, Neurohormones

## Abstract

In this study, the complex organization of the AnG in the giant freshwater prawn *Macrobrachium rosenbergii* was revealed using various techniques, including conventional histology, histochemistry, scanning electron microscopy, and X-ray tomography. The results showed the diversity of cells in the AnG and the detailed organization of the labyrinth’s tubule into four radiated areas from the central to peripheral zones. The study also demonstrated the expression of some vertebrate kidney-associated homolog genes, *aquaporin* (*AQP*), *solute carrier family 22* (*SLC-22*), *nephrin*, and *uromodulin*, in the AnG by qPCR. The result of in situ hybridization further showed the localization of *SLC-22* and *AQP* transcript in the bladder and labyrinth’s epithelium, specifically in regions 2, 3, and 4. Additionally, the study revealed neuropeptide expressions in the AnG by qPCR and in situ hybridization, i.e., *crustacean hyperglycemic hormone* (*CHH*) and *molt inhibiting hormone* (*MIH*), implying that the AnG may have a role in hormone production. Moreover, male and female prawns exhibited different levels of *AQP*, *SLC-22*, *nephrin*, and *CHH* expressions during the premolt and intermolt stages, suggesting a crucial role relevant to the molting stages. In conclusion, this study clarified the complex structure of the AnG in *M. rosenbergii* and demonstrated for the first time the expression of vertebrate kidney-associated genes and the possible endocrine role of the AnG. Further investigation is needed to clarify the role of these genes, particularly during ecdysis. The implications of these findings could significantly advance our understanding of the AnG in decapod crustaceans.

**Supplementary Information:**

The online version contains supplementary material available at 10.1007/s00441-024-03898-3.

## Introduction

Crustaceans have an excretory organ called the antennal gland (AnG), also known as the maxillary gland or green gland. This gland is located at the base of the antennae or maxilla (Lavalli and Spanier 2010) and is equivalent to the kidney in vertebrates (Wheatly 1985). In crustaceans, the AnG plays roles in water and mineral homeostasis, osmotic stabilization, ion regulation, and nitrogenous waste elimination (Buranajitpirom et al. 2010; Khodabandeh et al. 2005b, c; Tsai and Lin 2014). In addition, the AnG secretes and releases biomolecules with urine for chemical communication, especially related to male sexual behaviors (Atema and Cowan 1986; Bamber and Naylor 1997; Bose et al. 2017). It has recently been discovered that the AnG serves as a natural entry point for viruses and bacteria (De Gryse et al. 2020).

Structurally, AnG can also be viewed as mammalian nephrons in the kidney, which connect to a urinary bladder. The organization of the AnG is classically composed of four major regions: the coelomosac, labyrinth, nephridial canal, and bladder (Felgenhauer 1992; Khodabandeh et al. 2005a). Ultrafiltration takes place across the epithelium of coelomosac, which possesses podocyte endings on its basal laminar. Then, the filtrate empties into the different parts of the duct in the labyrinth and nephridial canal, where it undergoes modifications to produce urine. In fact, the structural organization of the AnG in crustaceans has been described in various species, including the crayfish *Procambarus clarkii* (Ueno and Inoue 1996), *Homarus gammarus* (Khodabandeh et al. 2005a), *Astacus leptodactylus* (Khodabandeh et al. 2005b), the mystacocarid *Derocheilocaris typica* (Hessler and Elofsson 2007), *Penaeus vannamei* (De Gryse et al. 2020), and *Macrobrachium rosenbergii* (Al-Mohsen 2009). However, the morphological and cellular organizations and the concept of its functions in relation to the kidney are not well established. Even though several studies have shown the localization of Na+-K+-ATPase and proposed the ion regulatory mechanism of AnG in various species of decapod crustaceans (Buranajitpirom et al. 2010; Khodabandeh et al. 2005b, c; Tsai and Lin 2014), no other kidney-related proteins have been shown for their expression and localization in the AnG.

Well-known vertebrate kidney-associated genes that are focused in this study include *aquaporin* (*AQP*), *nephrin*, *solute carrier family 22* (*SLC-22*), and *uromodulin* (Habuka et al. 2014; Fagerberg et al. 2014; Uhlén et al. 2015; Yu et al. 2015). Aquaporins are transmembrane proteins that play an important role in transporting water and solutes. They are found in almost all living organisms and are highly expressed in the kidneys, where they help regulate water reabsorption. *AQP1* is one of the genes that may be involved in osmoregulation in decapod crustaceans (Moshtaghi et al. 2016; Nash et al. 2022; Niu et al. 2020; Rahi et al. 2018). In the freshwater prawn *Palaemonetes argentinus*, AQP1 increases significantly during the premolt stage (Foguesatto et al. 2017). Nephrin is a protein found in podocyte pedicles that is crucial for ultrafiltration (Fukasawa et al. 2009; Pavenstadt et al. 2003). In crayfish, the coelomosac epithelial cells have many pedicles similar to vertebrate podocytes and function in urine filtration (Khodabandeh et al. 2005b). It has never been reported that nephrin is present in the AnG of crustaceans. Uromodulin is produced exclusively in the kidneys by renal tubular epithelium. It is evolutionarily conserved and has broader roles in kidney function, including regulating salt transport (Micanovic et al. 2020). The solute carrier 22 (SLC-22) is an organic ion transporter family that is highly abundant in human kidneys (Yee and Giacomini  2022). SLC-22 is also found in *Drosophila melanogaster*, suggesting its evolutionary conservation and importance for cellular function.

Interestingly, several reports have demonstrated the existence of neurohormones in the AnG, including crustacean hyperglycemic hormone (CHH), crustacean female sex hormone-like, glycoprotein alpha-2, pyrokinin, SIFamide (Chen et al. 2004; Nguyen et al. 2016), and molt inhibiting hormone (MIH) (unpublished data). Additionally, a key enzyme for methyl farnesoate degradation [juvenile hormone esterase-like (JHE-like) carboxylesterase (CXE)] was identified in the AnG of the Pacific white shrimp *P. vannamei* (Zhang et al. 2020). All these findings suggest the endocrine function of AnG.

Recently, it has been discovered that the caudal extension of AnG in *P. vannamei* fills with content volume that changes dynamically during molting, suggesting its role in the molting process (De Gryse et al. 2020). In addition, in the attempt to identify mating pheromones in female prawns, proteomic analysis of *M. rosenbergii*’s AnG has been conducted and revealed proteins with varying abundances across different molt stages (Bose et al. 2017). These data indicated that the functional role of AnG may differ between sexes and different molt statuses. Further investigation is needed to understand the implications of these findings.

Due to its biological characteristics, global distribution, and economic importance, the freshwater prawn *M. rosenbergii* has the potential to become a prominent model for the study of crustacean physiology and molecular biology research. In this study, we focused on the structure and expression of vertebrate kidney-associated homolog genes and some neurohormones in the AnG of *M. rosenbergii*, whose histological and gene expression information remains limited. Therefore, this study was conducted with two main objectives: (1) to provide a more detailed understanding of the basic organization of AnG based on morphology and histology; (2) to reveal the expression levels of vertebrate kidney-associated homolog genes in the AnG and provide information on the possible endocrine role of the AnG in *M. rosenbergii*. Considering the possible different functional roles of AnG between sexes and molt statuses, we also examined gene expression of AnG in males and females in premolt and intermolt stages. The organization of AnG was displayed in detail using traditional light and scanning electron microscopy and advanced synchrotron X-ray tomographic microscopy (SR-XTM). The culmination of these results unveiled several novel insights that have not been previously reported. Furthermore, the expression levels and localization of vertebrate kidney-associated homolog genes (*aquaporin* (*AQP*), *nephrin*, *solute carrier family 22* (*SLC-22*), and *uromodulin*) and some neurohormone-encoded genes (*CHH* and *MIH*) were demonstrated in different sexes and molting statuses of *M. rosenbergii*. This work may provide valuable information for future research on the structure and functions of the AnG in decapod crustaceans.

## Materials and methods

### Animals and tissue collection

Mature male and female *M. rosenbergii* (*n* = 10, each) were purchased from a local farm in Suphanburi Province, Thailand. For morphological and histological analysis, AnGs were collected and either fixed in Davison’s fixative solution or 2.5% glutaraldehyde and 2% paraformaldehyde in phosphate buffer (PB). For gene expression analysis, male and female AnGs were collected during the intermolt or premolt stage (*n* = 5 prawns/group), then immediately frozen at – 80 °C. The molt stages were determined based on the observations described by Kamaruding et al. (2017). Animal ethics was approved by the Experimental Animal Ethics Committee, Faculty of Science, Mahidol University, Thailand (MUSC61-022–424).

### Morphological observations using scanning electron microscopy (SEM)

The head and cephalothorax of the male prawn were dissected, and the AnG was photographed by a digital camera for gross morphological demonstration. Glutaraldehyde fixed AnGs were prepared for SEM using a method described by Pattarayingsakul et al. (2019) and Pudgerd et al. (2019). The fixed tissues were cut in the horizontal and sagittal planes, washed with 0.1 M PB, and incubated in 1% osmium tetroxide (OsO_4_) in 0.1 M PB at 4 °C for 2 h for post-fixation. Then, the tissue pieces were gently washed with water three times for 15 min each and dehydrated in increasing concentrations of ethanol (30% to 100%) two times each at 4 °C. The tissues were dried in a critical point-drying machine (Hitachi HCP-2) in liquid CO_2_. Then, they were placed and mounted on stubs with conductive carbon tape and consequently coated with platinum and palladium using a Hitachi E-120 ion sputter. The coated tissues were observed and photographed under a Hitachi scanning electron microscope S-2500.

### Synchrotron X-ray tomographic microscopy (SR-XTM)

Glutaraldehyde fixed AnGs were prepared following the same tissue preparation for SEM but without metallic coating steps. After the tissues were dried in a critical point-drying machine, they were subjected to X-ray tomography examination at the Synchrotron Light Research Institute (Public Organization), Nakhon Ratchasima, Thailand. Synchrotron radiation X-ray tomographic microscopy (SR-XTM) was performed at Beamline 1.2 W operated at 1.2 GeV. For prevention of sample movement during scanning, the tissue was fixed into Kapton tape with a cotton pad before mounting on the goniometer stage. In this case, the 350-micron thickness of the aluminum foil with a mean energy of 11.5 keV was attenuated to minimize artifacts such as ring artifacts. In addition, the 200-micron thickness of the YAG:Ce scintillator (Crytur, Czech Republic), lens-coupled X-ray microscope (Optique Peter, France), and sCMO camera (pco. edge 5.5, 2560 × 2160 pixels, 16 bits) were employed to collect the X-ray radiographies from 0 to 180 with an angular increment of 0.1 degree. The data preprocessing, background normalization, and CT reconstruction were calculated by using Octopus Reconstruction software (Tescan, Gent, Belgium) (Vlassenbroeck et al. 2006). After that, the reconstructed images of the sample were rendered in 3D tomographic reconstruction by using Drishti software (Limaye 2012).

### Histological staining using hematoxylin and eosin (H&E)

Davison’s fixed AnGs were subjected to an automatic tissue processor for dehydration (Leica TP120, Leica Biosystem), embedded in paraffin, cut at 6–7 µm, and stained with standard Harris hematoxylin and eosin (H&E) staining (Kruangkum et al. 2013, 2015). Briefly, the AnG sections were deparaffinized three times in xylene, followed by gently dipping in decreasing dilutions of ethanol from 100 to 70% for rehydration. Then, all sections were quickly soaked in hematoxylin and activated in tap water. Sections were dipped in eosin for 2 min and then dehydrated in 80% to 100% ethanol. Finally, the sections were cleared in xylene and mounted in Permount® medium (Fisher Scientific). Tissue sections were observed under a light microscope (Leica DM750) and images were taken with a digital camera (Leica ICC50 HD).

### Histochemical staining using periodic acid Schiff’s reaction

Prepared AnG sections were consecutively selected for periodic acid-Schiff (PAS) staining. Sections were deparaffinized with xylene and rehydrated with serial decreasing concentrations of ethanol. All sections were transferred into a jar containing tap water for 5 min and then placed into 0.5% periodic acid for 5 min. Tissue sections were washed with tap water and immersed in Schiff’s reagent for 30 min. Subsequently, the sections were washed with tap water and counterstained with Mayer’s hematoxylin for 2 min. Finally, the tissue sections were dehydrated, cleared in xylene, and mounted in Permount® medium (Fisher Scientific). The tissue sections were observed under a light microscope (Leica DM750) with a digital camera (Leica ICC50 HD).

### Semithin sectioning and toluidine blue staining

Glutaraldehyde-fixed AnGs were processed for Epon embedding as previously described (Sriurairatana et al. 2014). The semithin Sects. (900 nm–1 μm) were cut by a Leica Ultracut UCT ultramicrotome (Leica, Wetzlar, Germany) and stained with toluidine blue. The sections were observed under a light microscope (Olympus BX51) with a digital camera (Olympus DP73).

### Identification of vertebrate kidney genes homolog in *M. rosenbergii* AnG

Bioinformatic analysis with tBLASTn (Bose et al. 2017) was used to search for *M. rosenbergii* homolog sequences of vertebrate kidney genes in the publicly available AnG transcriptome database (SRA number: PRJNA381087) (Table 1). Moreover, the nucleotide sequence of crustacean hyperglycemic hormone (CHH) (GenBank: AF219382.1) and molt inhibiting hormone (MIH) (GenBank: KC990939.1) specific to this species were obtained from the NCBI database.

**Table 1:** List of vertebrate kidney genes and homolog identified in *M. rosenbergii* AnG transcriptome

Protein in kidney function	*M. rosenbergii* transcript	BLAST hit and species	Databases	*E*-value
Uromodulin	CL1148.Contig1_MrAnG	Uromodulin [*Canis familiaris*]	SwissProt- annotation	1.00E − 24
Solute carrier family 22	Unigene16991_MrAnG	Solute carrier family 22 member 8 [*Sus scrofa*]	Nr-Annotation	8.00E − 10
Aquaporin	Unigene28443_MrAnG	Aquaporin-10 [*Homo sapiens*]	SwissProt- annotation	6.00E − 24
Nephrin	Unigene20598_MrAnG	Nephrin [*Mus musculus*]	SwissProt-annotation	3.00E − 13

### In situ hybridization

In situ hybridization was performed using DIG-labeled DNA probes, following a previously reported protocol (Chotwiwatthanakun et al. 2016) with some modifications. Briefly, the specific DNA probes for *AQP*, *SLC22*, and *CHH* were prepared with a DIG-PCR labeling Mix kit (REF 11585550910, Roche, Germany) and the specific primers (shown in Table 2). For preparation of the negative control probes, a similar protocol was performed without DIG labeling.

**Table 2:** List of specific primers used in this study and details

**Name of gene**	**Primer name**	**Nucleotide sequence** (5′–3′)	**Description**	**Amplicon size (bp)**
*Nephrin*	NephrinF	AGGAACGAGTGTGTGGTCGAA	RT-PCR/ qPCR	332
	NephrinR	TACCACCGTCTTGTATGGCG		
*SLC22*	SCF22F	CGCTTCACGAGTAGGGTCAA	RT-PCR/ qPCR/ in situ probe synthesis	302
	SCF22R	ACGACTGTGGTTGAGGGAAC		
*AQP*	AQF	CTCTCTTCGTCGGCTTCACC	RT-PCR/ qPCR/ in situ probe synthesis	221
	AQR	GTATATCGCCACGCCCAAGA		
*Uromodulin*	UromoF	CTGGTTACTCCGGTGATGGG	RT-PCR	709
	UromoR	AGGCAAGGCACTTTGTGAG		
*CHH*	CHH-F	CCTCTCATCTGCCGCTTTGT	RT-PCR/qPCR	112
	CHH-R	CCTATCTGGAGTTGAGCGCC		
	CHH2-F	GCCCAAGCAACCGTCAAAAA	RT-PCR in situ probe synthesis	779
	CHH2-R	GACGAAAGAACAACAGCGCA		
*MIH*	MIH1-F	CCAGACAACGCAAGGGATCT	RT-PCR/qPCR	165
	MIH1-R	TCGTCGCATACCCTGACAAC		
	MIH2-F	AACCAGACAACGCAAGGGAT	RT-PCR	381
	MIH2-R	CGTGTGGTCCTGGAGCTTAT		
*16 s rRNA*	16sRNA-F	TGACCGTGCRAAGGTAGCATA	RT-PCR/qPCR	153
	16sRNA-R	TTTATAGGGTCTTATCGTCCC		

The concentration of DIG-labeled DNA probes used for hybridizing buffer preparation was approximately 100 ng/μl. Paraffin sections of AnGs were deparaffinized in xylene, rehydrated with decreasing concentrations of ethanol (100% to 70%), and immersed in 1 × TNE buffer (500 mM Tris–Cl, 100 mM NaCl, l0 mM EDTA, pH 7.4). Tissue sections were treated with 5 μg/ml of proteinase K for 15 min at 37 °C, washed in TNE buffer and distilled water for 10 min, and then postfixed with cold 4% paraformaldehyde for 5 min. The tissues were washed with distilled water for 5 min before being treated with 20% ice-cold acetic acid for 20 s and washed with distilled water for 5 min. All tissue sections were equilibrated with prehybridization buffer containing 4xSSC (0.6 M NaCl and 0.06 M Na-citrate) and 50% (v/v) deionized formamide for 30 min at 37 °C. The hybridization buffer (containing 50% deionized formamide, 50% dextran sulfate, 50 × Denhardt’s solution [Sigma Chemical Co.], 20 × SSC, 10 mg/ml salmon sperm DNA [Invitrogen]) containing probes and nonprobes was preheated at 95 °C before immediately chilling on ice. The probes and nonprobes were applied to the tissue sections and covered with glass coverslips to prevent evaporation. The tissues were incubated in humidified chambers at 42 °C overnight. After incubation, the coverslips were removed from the tissue sections. The tissues were washed with 2 × SSC at 37 °C, 1 × SSC at 42 °C for 10 min, and 0.5 × SSC at 42 °C for 10 min and then placed in buffer I (1 M Tris–HCl, 1.5 M NaCl) for 5 min. The tissues were incubated with buffer II (buffer I contained a blocking reagent (Roche, Germany)), and alkaline phosphatase-conjugated anti-digoxigenin antibody (1:500 in buffer II) was applied for 4 h at 37 °C. After that, the tissue sections were washed twice in buffer I for 10 min each at room temperature. The sections were placed in buffer III (detection buffer containing 100 mM Tris–HCl, 1.5 M NaCl, 50 mM MgCl_2_6H_2_O), and then, nitro blue tetrazolium-5-bromo-4-chloro-3-indolyl-phosphate (NBT-BCIP) substrate was added into the sections until the signal was detected. The sections were mounted with glycerol buffer (1:9 PBS and glycerol) and covered with coverslips for observation under a microscope equipped with a digital camera. Nuclear counterstaining was performed using Bismarck Brown Y (Sigma®, Germany).

### RNA extraction, cDNA synthesis, and PCR (RT-PCR and qPCR)

Antennal glands were homogenized in TRI reagent (TRI Reagent®, Molecular Research Center, Inc.). RNA extraction was performed according to the conventional protocol. Briefly, the tissue homogenate was incubated in chloroform for 10 min and centrifuged. The supernatant containing total RNA was collected, precipitated by isopropanol, and finally washed with ethanol twice. The RNA pellets were dried at room temperature before being dissolved in RNase-treated DEPC water. The quantity and quality of extracted RNAs were measured and evaluated using a Nanodrop One spectrophotometer (Thermo Fisher Scientific USA). The RNA integrity was assessed by agarose gel electrophoresis. One hundred nanograms of total RNA was pretreated with DNase I (Thermo Scientific) to eliminate genomic DNA contamination. cDNA synthesis was performed using a RevertAid First-Strand cDNA synthesis kit (#K1612, Thermo Scientific) following the manufacturer’s protocol. cDNAs were stored at − 20 °C until use.

Semi-quantitative RT‒PCR was performed by using a pooled cDNA sample of each group as a template. The amplification protocol was performed according to the manufacturing protocol using KAPA2G Fast HotStart ReadyMix with dye Tag-polymerase (Kapa Biosystems). The specific primers used in this study are listed in Table 2. PCR conditions were as follows: 94 °C for 3 min; 94 °C for 15 s; 28 cycles of 60 °C for 10 s and 72 °C for 45 s for amplification of *AQP*, *SLC22*, uromodulin, *CHH* and *MIH* genes; and 35 cycles for the nephrin gene. Finally, 72 °C for 5 min was performed for a final extension. *16 s rRNA* gene was used as the internal control, while the negative control represented no cDNA use. PCR products were examined with agarose gel electrophoresis, stained with ethidium bromide, and visualized using a Geldoc (Syngene, Frederick, MD).

For qPCR analysis, the cDNA of each sample, except nephrin transcript, was diluted 10 times. A master mix containing 0.6 μl of specific primer mixture of each gene and 2 × master mix of SYBR Green buffer (iTaq Universal SYBR® Green Supermix, Bio-Rad Laboratories, Inc.) was prepared, and qPCR was performed according to the recommended protocol (Bio-Rad Laboratories, Inc.). Briefly, triplicate qPCR reaction samples were optimized for the analysis. Specific primers used in this study are listed in Table 2. The qRT-PCR reactions were performed under the monitoring of the Bio-Rad iCycler™ machine (Bio-Rad, Hercules, CA, USA) with the following conditions: 95 °C for 30 s and 40 cycles of 95 °C for 5 s followed by 60 °C for 30 s and finally 65 °C for 5 s followed by 95 °C for 50 s for melting temperature analysis. 16 s rRNA was subjected to internal control and normalization. The analysis was calculated based on the equation shown below.

$$Ratio\;(test/calibrator)\;=\;2\Delta CT,\;where\;\Delta CT=CT\;(calibrator)\;-\;CT\;(test)$$

The results after being calculated and calibrated with the reference were reported in mean ± S.E. For the statistical analysis, mean ± S.D. was used and analyzed by GraphPad Prism and SPSS software.

## Results

### Gross anatomy and morphology of the antennal gland in *M. rosenbergii*

**Fig. 1:**
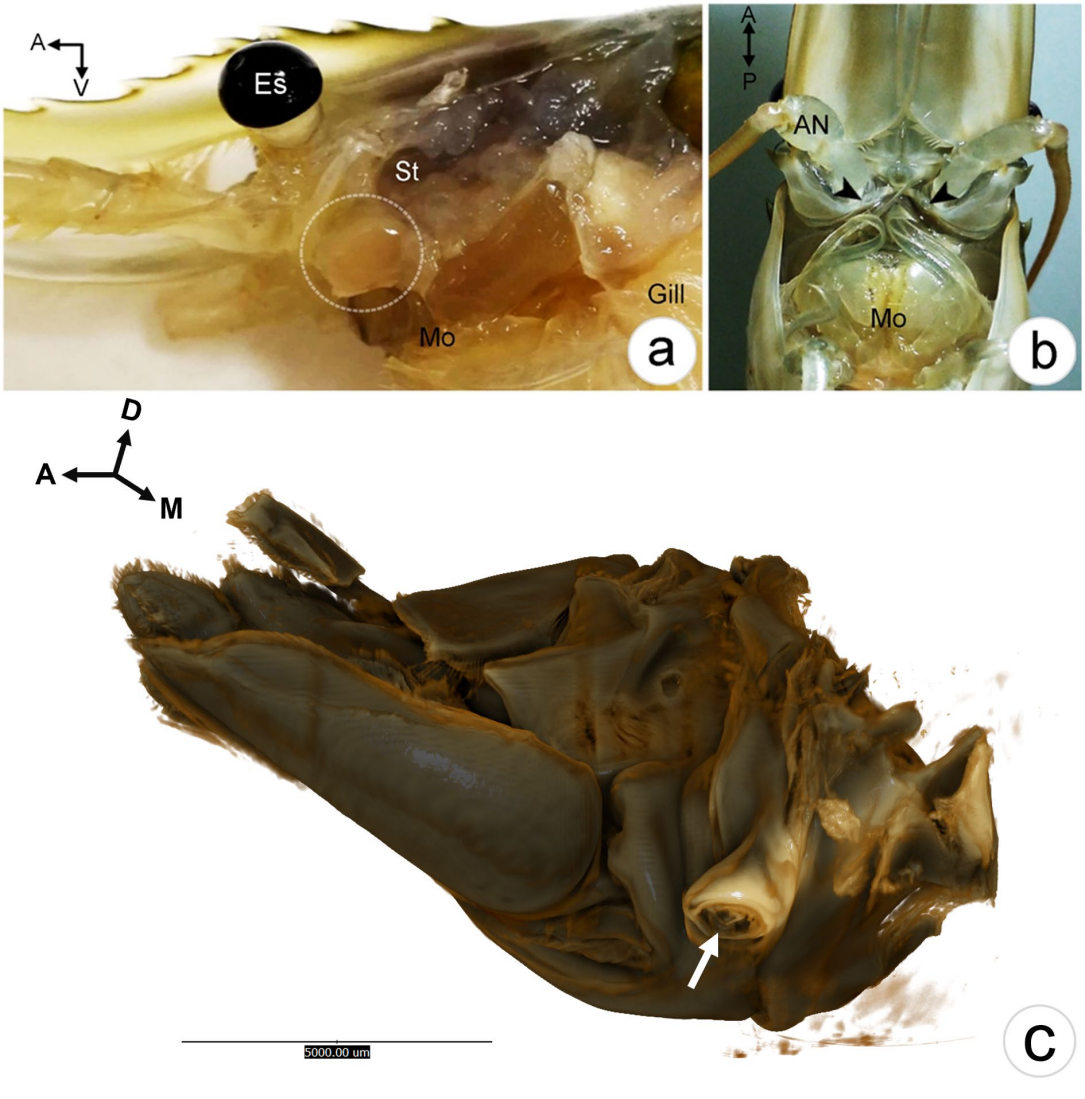
Photographs showing the AnG and the nephropore of *M. rosenbergii*. **a** A round yellowish mass of AnG (circle) is located inside the base of the antenna. **b** A pair of nephropores is observed at the base of the antenna (arrow heads), close to the mouth part (Mo). **c** A nephopore (arrow) at the right antenna base is observed by SR-XTM. Abbreviations: *A*, anterior; *AN*, antenna base;* D*, dorsal; *Es*, eyestalk; *M*, medial; *Mo*, mouth part;* P*, posterior; *St*, stomach; *V*, ventral

The AnG of adult prawn *M. rosenbergii* (male and female) appeared as a round yellowish organ located underneath the cuticular plate of the maxilla part and dorsolateral to the mouthparts (Fig. 1a). The gland was surrounded by tight bundles of connective tissue and muscle inside. Externally, there was a pair of tubercular protrusions of the nephropore or excretory pore, and the opening of the AnG appeared at the base of the antenna (Fig. 1b). SR-XTM showed nephropore protrusion at the base of the right antenna in the medial view (Fig. 1c).

### Structural organization of the antennal gland observed by SR-XTM

**Fig. 2:**
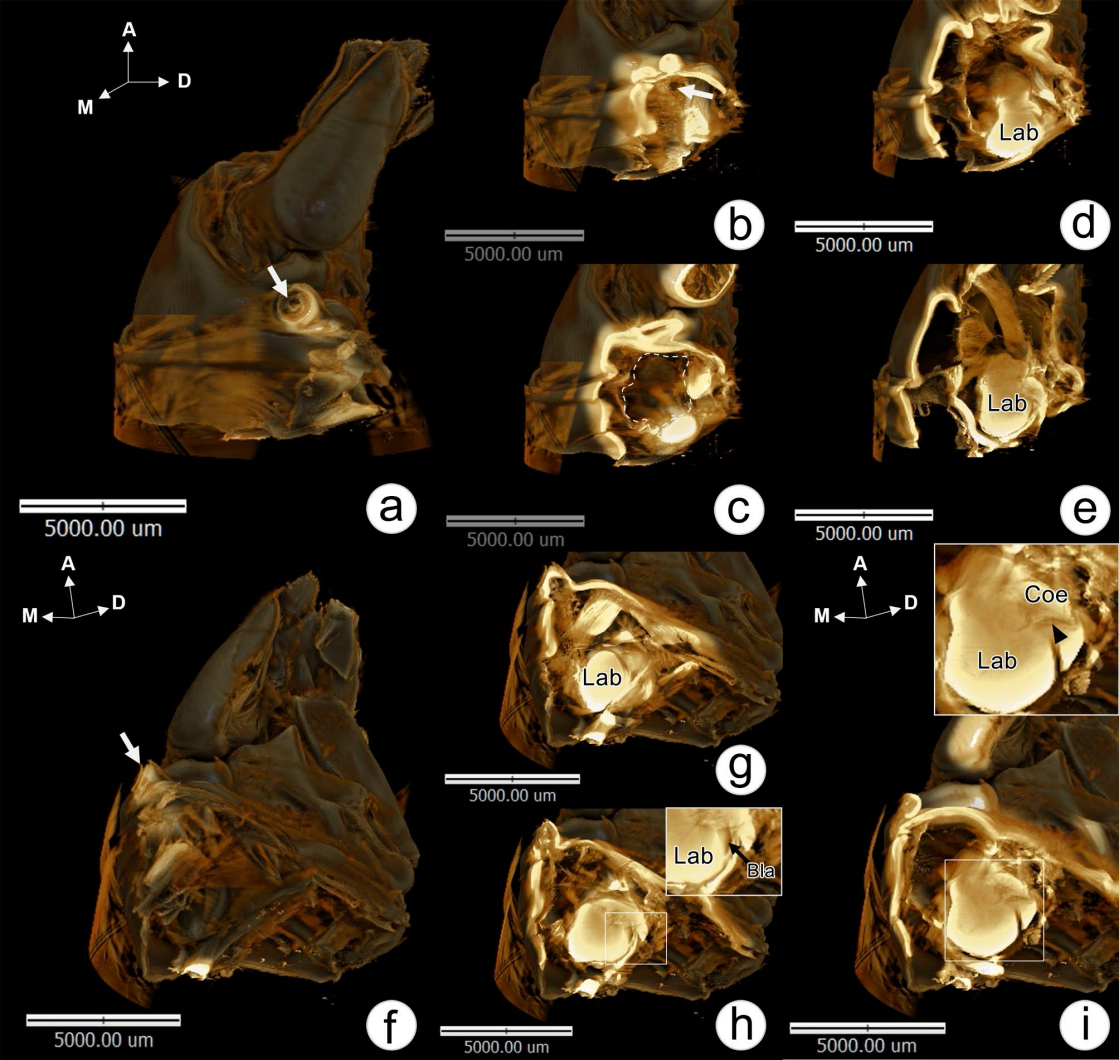
Single optical sections of the AnG by SR-XTM. **a**–**e** Single optical sections, from the most medial to lateral side, of the base of the right antenna show an opening of a nephropore (arrow, **a**), a uronephric duct (arrow, **b**), a chamber of the bladder (dotted line, **c**), and the labyrinth (Lab). **f**–**i** Serial-section tomographic photographs at the base of the antenna in the posterior-medial view show the structural organization of the bladder (Bla), labyrinth (Lab), and coelomosac (Coe). Part of the Bla extends into the margin of the Coe and Lab (arrow, h). An antennary artery enters the Coe (arrowhead, inset in i)

**Fig. 3:**
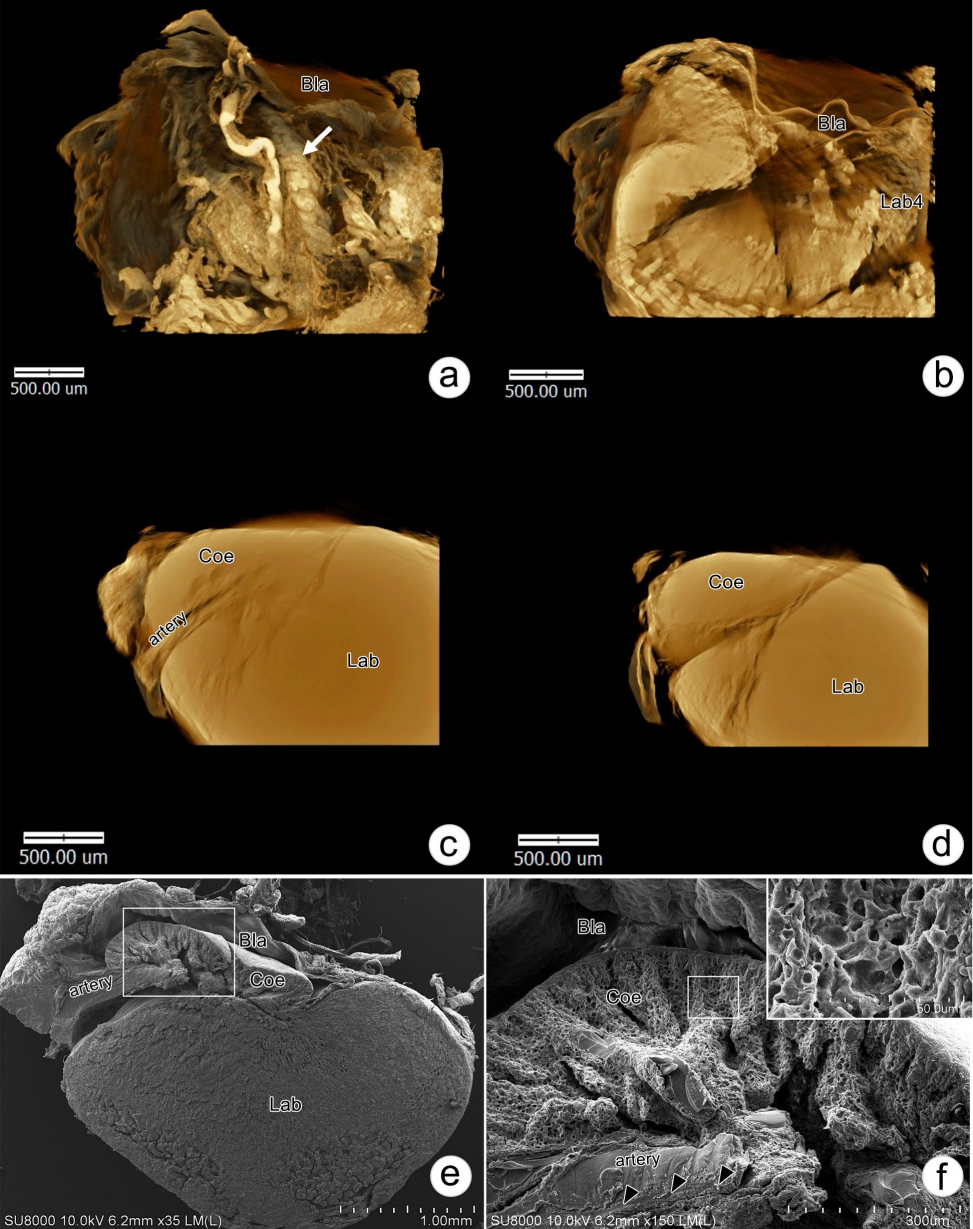
Photographs of AnG by SR-XTM and scanning electron microscopy (SEM). **a**–**d** Tomographic photographs of an isolated AnG show a structure of coiled ductules (**a**, arrow) interconnecting to the bladder (Bla), a hemal space in labyrinth 4 (Lab4) (**b**), and an opening of the artery into the coelomosac (Coe) (**c**, **d**). **e**, **f** Scanning electron micrographs of a bisected AnG show the organization of the AnG and the opening of the antennary artery in the Coe (**e**). **f** Coe stromal cells show a sponge-like structure (**f**, inset), and podocytes are associated with the artery (arrowheads)

Single optical sections of the right antenna from the medial to lateral aspect (Fig. 2a, b, c, and Supplement 1) and posteromedial view (Fig. 2f and Supplement 2) were provided by X-ray tomography. A nephropore was located at the ventromedial side of the antenna (Fig. 2a, arrow) and communicated internally with an empty uronephric duct (Fig. 2b, arrow), which continuously opened into a large space of the urinary bladder surrounded by a hemal sinus (Fig. 2c). Parasagittal-section images of the AnG showed the structure of the labyrinth (Lab), which is located ventromedially to the coelomosac (Coe) (Fig. 2d, e). Posteromedial views of the longitudinal sections of nephropore and uronephropore duct (Fig. 2f) revealed the relationship of Lab, Coe, and bladder (Bla) (Fig. 2g–i). The Lab was located posteromedial to the Coe (Fig. 2g) and was closely associated with the Bla, which appeared on the medial side. Moreover, part of Bla was also found to extend into the margin of the Coe and Lab (Fig. 2h). A parasagittal section of the AnG at the region of Lab and Coe showed the invagination of an antennary artery into the Coe (Fig. 2i). Higher magnification of an isolated AnG showed coiled ductules connecting Lab4 and Bla (Fig. 3a). A cross-section of the AnG from the ventral to dorsal part showed a hemal space located in Lab4 (Fig. 3b). The antennary artery was found to project into the Coe sinusoidal space (Fig. 3c). The Coe appeared as a looping tubule located on the dorsolateral side of the AnG (Fig. 3d). With SEM, a longitudinal section of AnG revealed the relationship of the Lab, Coe, and Bla (Fig. 3e). The cellular organization of Coe appeared as a sponge-like structure (Fig. 3f, inset). Filamentous materials were found to cover the outside of the antennary artery in the Coe (Fig. 3f, arrowheads).

### Histological appearance of the antennal gland

**Fig. 4:**
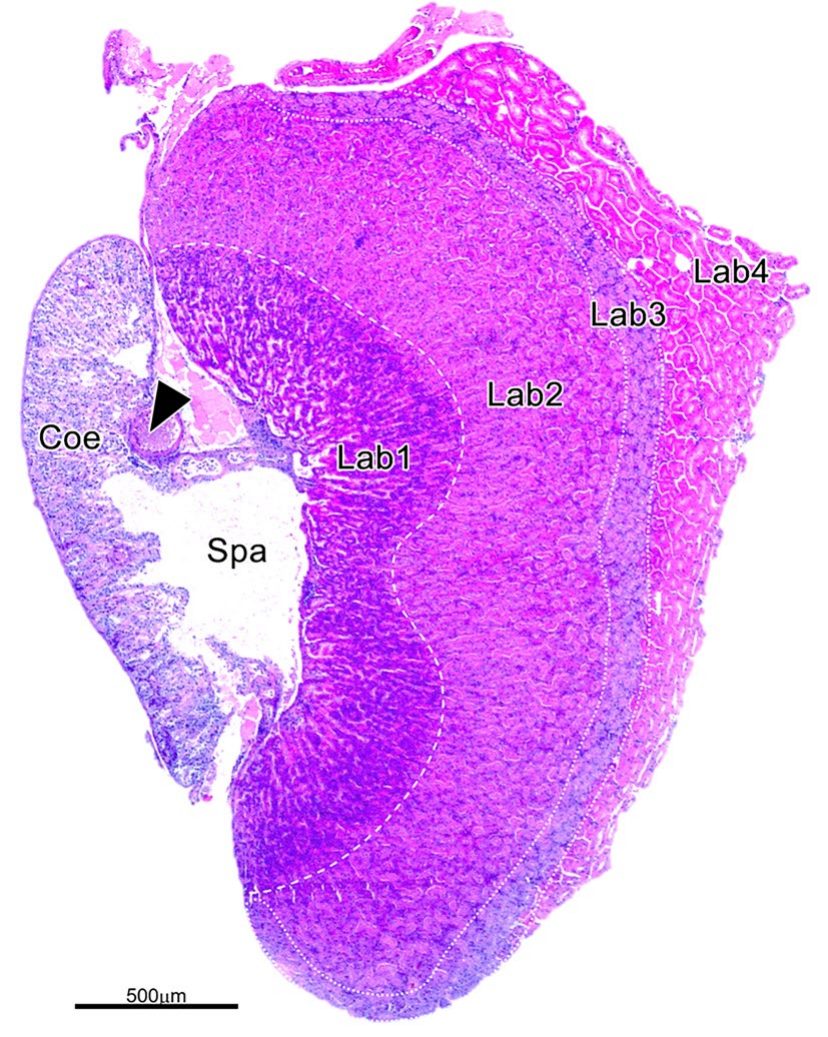
Histological micrograph of an AnG stained with hematoxylin and eosin (H&E). Four differential zones of the labyrinth (Lab) are presented based on the H&E staining properties of the cells in each zone

Histological analysis of AnG was performed from H&E-stained sections. A longitudinal section of AnG showed a large antennary artery (Fig. 4, arrowhead) situated in the Coe. A large coelomosac sinusoidal space (Spa) was observed between the Coe and Lab; this space apparently carries filtrates from the Coe and directs them to the Lab. The Lab could be divided into four major regions based on the acidophilic and basophilic properties of the H&E-stained cells. Most of the epithelial cells lining the tubules in Lab1 showed typically deep blue-purple stained cells. The cells lining the tubules in Lab2 contained mostly eosinophilic cytoplasm; this region occupied half of the total area of the AnG. The thin region of Lab3 had lighter H&E staining than the other Lab regions. Lab4, located in the most peripheral part of AnG, showed strong eosinophilic-cytoplasmic staining of tubular cells (Fig. 4).

**Fig. 5:**
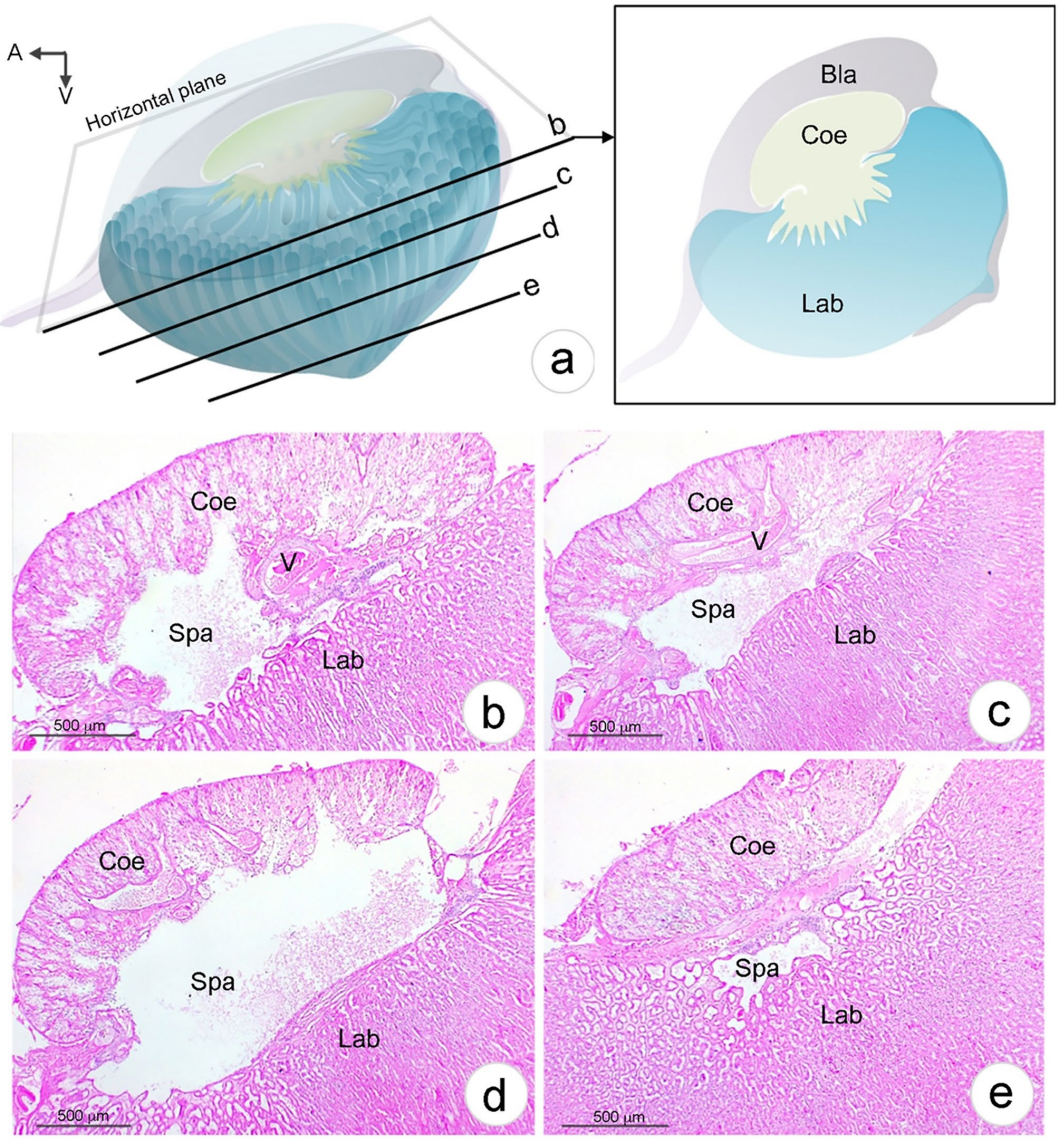
Schematic illustration of the antennal gland (AnG) structure and coelomosac (Coe) histology. **a** Schematic diagram of the AnG showing sectional planes of the images in panels b–e. **b**–**e** H&E-stained Coe midsagittal to parasagittal sections. Abbreviation: *Lab*, labyrinth; *Spa*, coelomosac sinusoidal space; *V*, vessel (antennary artery

Horizontal sections of Coe were observed at four different levels (Fig. 5a). A large antennary artery entered the Coe (Fig. 5b) and then branched into smaller vessels in the septa of the Coe (Fig. 5c, d). Most of the Coe was interconnected to Lab1 via a single large space called the coelomosac sinusoidal space (Spa). However, we observed part of the bladder chamber containing urine in the inter-peripheral space between the Coe and Lab (Fig. 5e).

### Cellular components of the antennal gland based on semithin sectioning and PAS staining

**Fig. 6:**
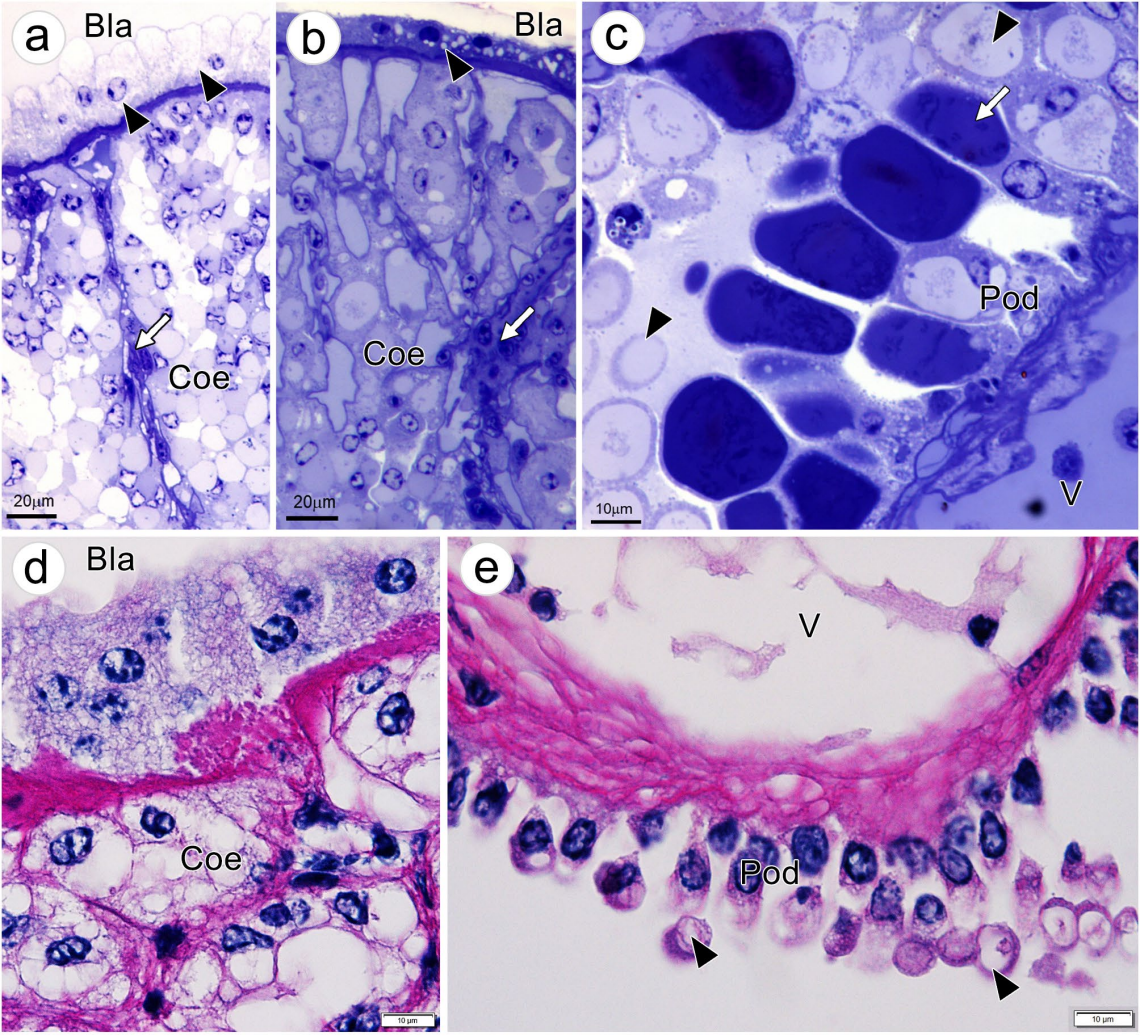
Photographs of semithin sections and PAS staining of the coelomosac (Coe). **a**, **b** Coe stroma contains irregularly shaped cells and mesangial-like cells in the Coe septa (white arrow). The Coe is lined outside with epithelial cells of the bladder (Bla), columnar- or cuboidal-shaped cells (arrowhead in a and b); they share basal laminar with each other. **c** Podocyte-like cells (Pod) attach to the basal lamina of the vessel (V) and contain large cytoplasmic vacuoles; many cytoplasmic blebs (arrowhead) appear. Several vacuoles contain materials densely stained with toluidine blue (white arrow), whereas some appear empty with dispersed materials. **d** Strong PAS staining is observed in the basal lamina of Bla’s epithelial cells and the Coe. **e** PAS staining is intense in the vascular wall and cytoplasmic blebs of the Pod (arrowheads). Abbreviation: *V*, vessel

Semithin sections of the AnG stained with toluidine blue revealed more details of the cellular components of AnG. Epithelial cells of Bla were columnar or cuboidal in shape (Fig. 6a, b, arrowhead) and are closely associated with the Coe, as they share basal laminar together. The Coe stroma contained irregularly shaped cells loosely organized in a space separated by Coe septa (Fig. 6a, b, arrow). Mesangial-like cells were present in the septa (Fig. 6a, b, arrow). The antennular vessel was found to be surrounded by processes of podocyte-like cells (Pod) at the lower margin of Coe (Fig. 6c); smaller branches of the vessel appeared throughout the septa (data not shown). The processes of Pod attached to the basal lamina of the vessel, whereas the apical side of the Pod presented large vacuoles containing materials densely stained with toluidine blue and appeared as cytoplasmic protrusions (Fig. 6c, arrow). Some vacuoles appeared empty while others having dispersed materials inside (Fig. 6c). Several cytoplasmic protrusions of Pod, called “blebs,” were apparently pinched off as apocrine-like secretory patterns (Fig. 6c, arrowhead). Strong PAS staining was observed in the basal lamina of Bla’s epithelial cells and Coe (Fig. 6d), the vascular wall, and cytoplasmic blebs of the Pod (Fig. 6e, arrowheads), suggesting glycoprotein contents in these structures.

**Fig. 7:**
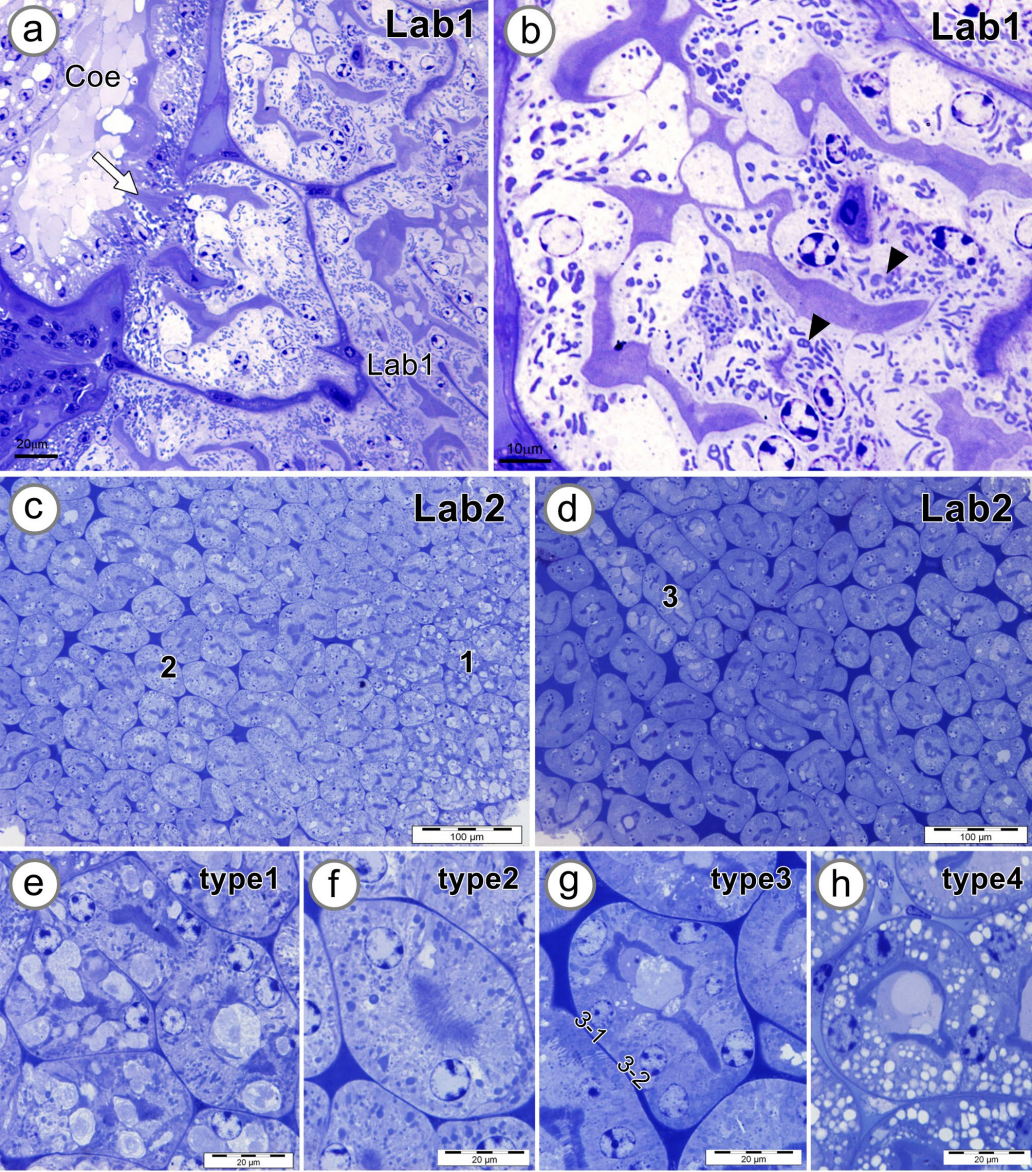
Photographs of semithin sections showing the zone 1 and zone 2 labyrinth (Lab1, Lab2). **a** Lab1 communicates with the coelomosac (Coe) space via an opening indicated by the arrow. **b** Tubular cells of Lab1 contain numerous mitochondria (arrowheads). **c**, **d** Low magnification of Lab2 presents cross-sections of many tubules, which can be divided into four types based on cellular characteristics. **e** Epithelial cells of type 1 tubules contain large vacuoles and numerous mitochondria. **f** Epithelial cells of type 2 tubules contain numerous mitochondria and apical brush borders without large vacuoles. **g** Tubular epithelial cells of type 3 tubules can be divided into two types, type 3–1 or dark cells, and type 3–2 or light cells. **h** Type 4 tubular epithelium contains a large number of small clear vacuoles in the cytoplasm

Semithin sections of the four regions of Lab were examined. Lab1 communicates with Coe via the Coe space (Fig. 7a). Filtrates apparently pass through the luminal area of Lab1 tubules via ductules (Fig. 7a, arrow). The tubules in Lab1 had an irregular shape, branching, and a wide lumen filled with filtrates. The tubular epithelial cells showed a concentric nucleus, long apical brush borders, and numerous mitochondria (Fig. 7b, arrowheads). The Lab2 zone showed numerous tubular structures occupying the major area of Lab (Fig. 7c, d). The semithin section of the Lab2 area revealed four types of tubules according to the cellular characteristics of the tubules (Fig. 7e-h). The epithelial cells of type 1 tubules showed a single layer of cuboidal cells containing large cytoplasmic vacuoles, numerous mitochondria, and an apical brush border (Fig. 7e). No large vacuoles were observed in the simple cuboidal epithelium of type 2 tubules; the tubular cells contained numerous mitochondria and apical brush borders (Fig. 7f). In the type 3 tubule, the single layer of epithelial cells with apical brush borders could be distinguished into two types based on the staining intensities, e.g., type 3–1 or dark cells and type 3–2 or light cells showing basally located mitochondria (Fig. 7g). The simple cuboidal epithelium of type 4 tubules contained numerous small vacuoles, likely lipid droplets that were dispersed throughout the cytoplasm (Fig. 7h).

**Fig. 8:**
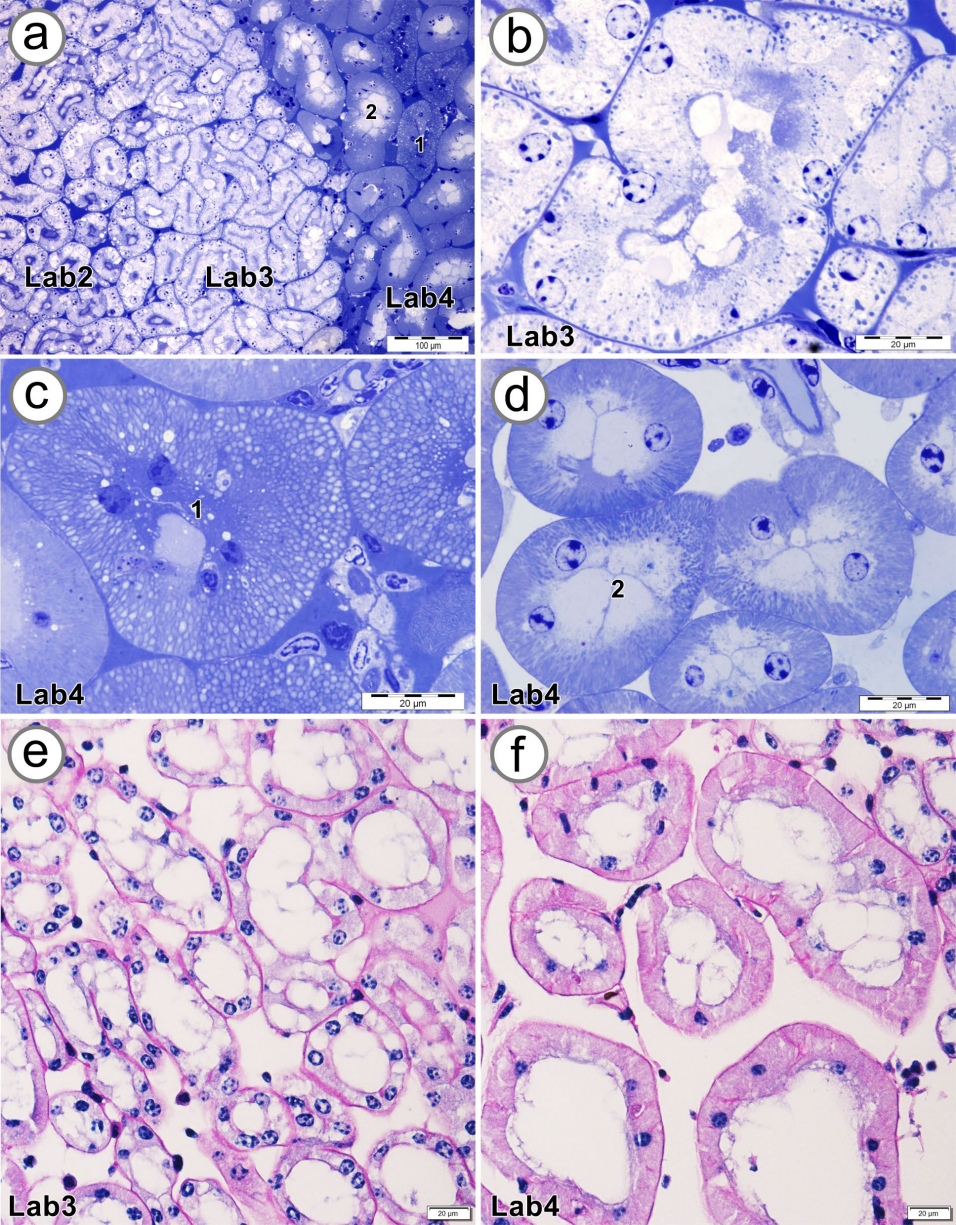
Photographs showing semithin sections and PAS staining of zones 2–4 of the labyrinth (Lab2, Lab3, and Lab4). **a** Overview of Lab2, Lab3, and Lab4; two types of Lab4 tubules are labeled. High-magnification images of Lab3 (**b**), type 1 and type 2 tubules of Lab4 (**c** and **d**, respectively), and PAS staining of Lab 3 and 4 (**e** and **f**, respectively)

Histological examination showed that Lab3 connects Lab2 and Lab4 (Fig. 8a). The tubular epithelial cells of Lab 3 showed pale stained cytoplasm, basally located mitochondria, and basally located nuclei (Fig. 8b). The tubular epithelial cells of Lab4 could be divided into 2 subpopulations, types 1 and 2. Type 1 showed apically located nuclei and numerous small vacuoles (Fig. 8c). Type 2, the dominant population in Lab4, presented pale cytoplasmic protrusions bulging into the luminal area, concentric nuclei, and basally located mitochondria with basal membrane infoldings (Fig. 8d). The PAS-stained section of Lab3 revealed prominent PAS-positive material at both apical and basal lines (Fig. 8e), whereas the Lab4 epithelial cells showed PAS-positive material in the cytoplasm and at the basal lamina (Fig. 8f).

### Expression of AQP, SLC22, nephrin, and uromodulin in the antennal gland

**Fig. 9:**
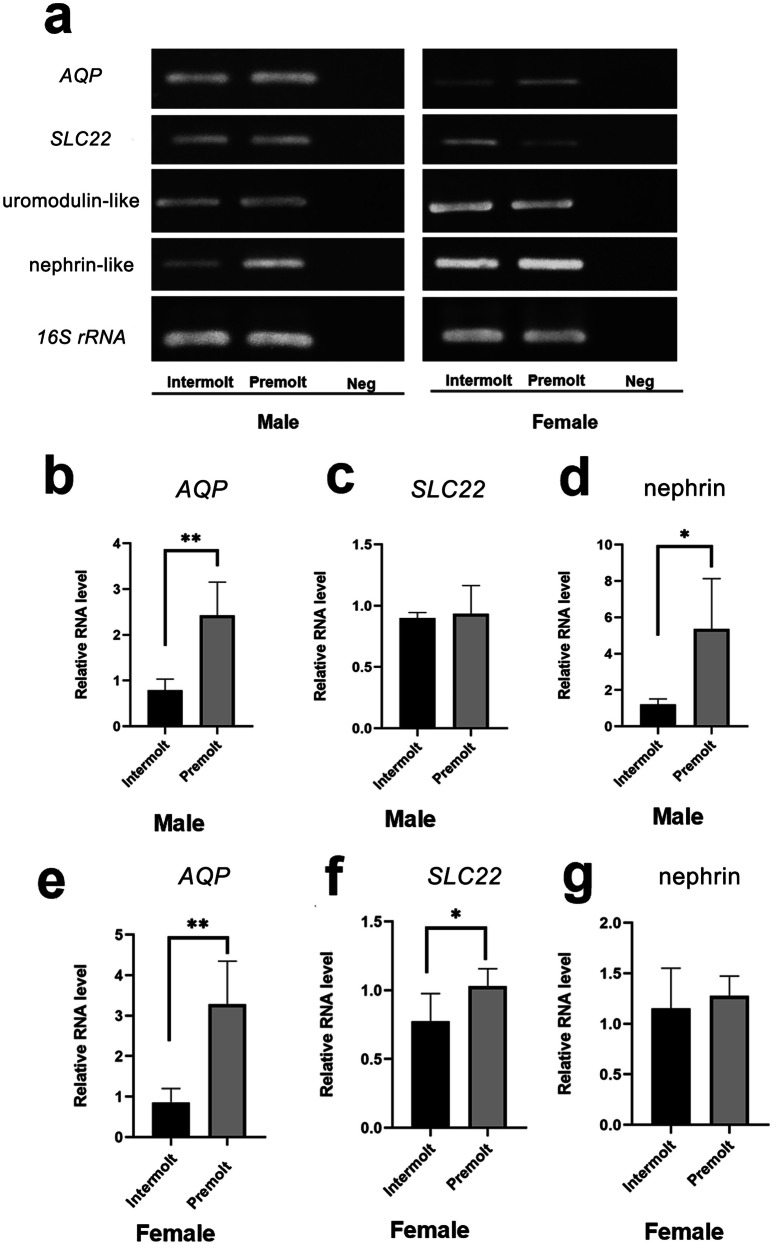
Expression of osmoregulatory genes, aquaporin (*AQP*), solute carrier family *22 (SLC22)*, uromodulin-like, and nephrin-like, in the AnG of males and females during the intermolt and premolt stages. **a** RT-PCR analysis of the four osmoregulation genes; 16S rRNA was used as an internal control. **b**–**g** Relative expression of the *AQP*, *SLC22*, and nephrin-like transcripts in the AnG of males (**b**–**d**) and females (**e**–**g**) during intermolt and premolt stages. * and ** indicate significant differences at *p* ≤ 0.05 and *p* ≤ 0.01, respectively

RT-PCR and quantitative PCR techniques were used to determine the expression of *AQP* and *SLC22*, *nephrin-like*, and *uromodulin-like* genes, during intermolt and premolt stages in male and female prawns. The expression of *AQP* was high in the premolt stage in both male and female prawns (Fig. 9a). The relative expression of *AQP* was 2- and threefold higher in the premolt stage than in the intermolt stage in males and females, respectively (Fig. 9b, e). The expression of *SLC22* was high in females during the premolt stage (Fig. 9a, c, f). In addition, the expression of the nephrin-like gene was significantly upregulated approximately fivefold in males during the premolt stage (Fig. 9a, d, g). However, the relative expression level of the uromodulin*-*like transcript was not significantly different between males and females in both the intermolt and premolt stages (Fig. 9a).

**Fig. 10:**
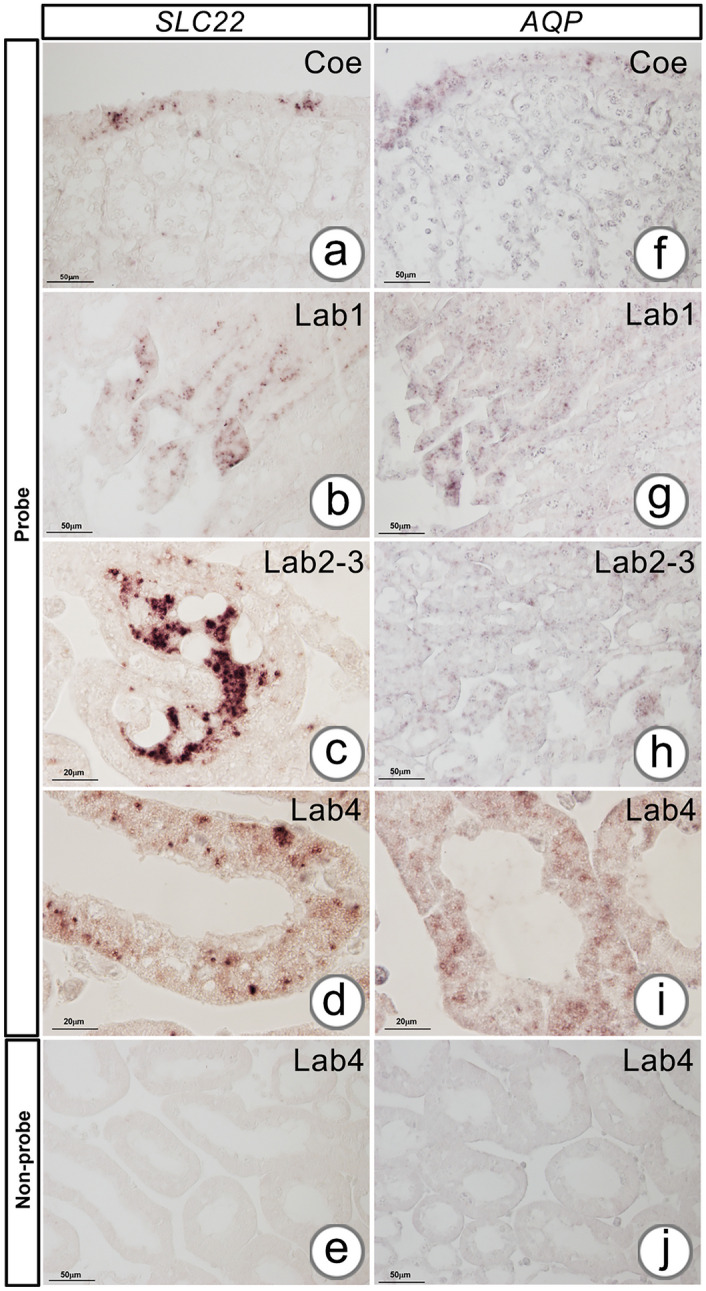
In situ hybridization of soluble carrier protein *22* (*SLC22*) and aquaporin (*AQP*) transcripts in the AnG. The positive signals of *SLC22* (**a**–**d**) and *AQP* (**f**–**i**) present in the Coe (**a**, **f**), Lab1 (**b**, **g**), interjunction of Lab2-3 (**c**, **h**), and Lab4 (**d**, **i**). The negative controls of *SLC22* and *AQP* are shown in e and j, respectively

In situ hybridization showed a similar pattern of *SLC22* and *AQP* expression in AnG. The *SLC22* transcript was specifically localized in Bla’s epithelial cells, which are located close to the Coe (Fig. 10a), and the epithelial cells of Lab2 tubules, which are connected with the coelominal sinusoidal space (Fig. 10b). In addition, the *SLC22* transcript was predominantly expressed in the apical site of tubular epithelial cells in Lab2 connecting to Lab3 (Fig. 10c) and in the tubular epithelial cells of Lab4 (Fig. 10d). Similarly, the *AQP* transcript was distributed in Bla’s epithelial cells, Coe cells, and podocytes (Fig. 10f). Moreover, the *AQP*-positive signal was found in the tubular epithelial cells of Lab2 (Fig. 10g), the tubular epithelial cells in Lab2 connecting to Lab3 (Fig. 10h), and the cells of Lab4 (Fig. 10i). No positive signals were observed in the negative control where the probes without DIG labeling were used (Fig. 10e and j).

### Expression of CHH and MIH genes in the antennal gland

**Fig. 11:**
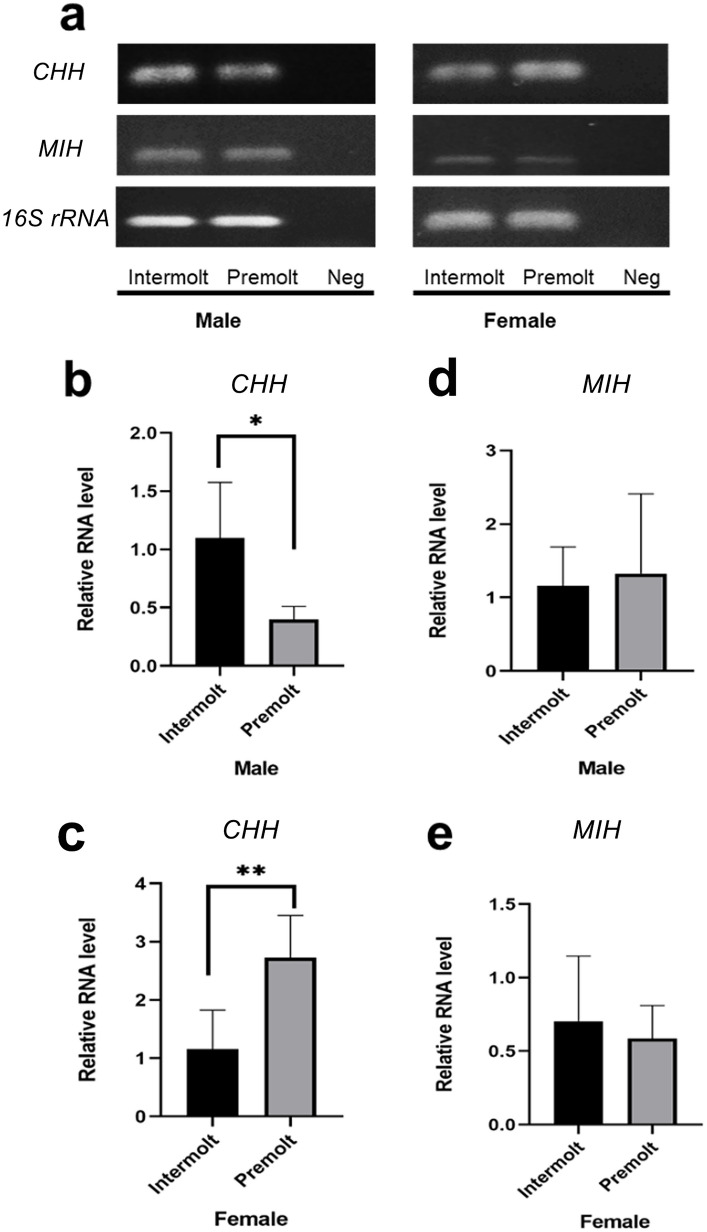
Expression of *CHH* and *MIH* transcripts in the AnG of males and females during the intermolt and premolt stages. **a** RT-PCR analysis of *CHH* and *MIH* expression in AnG; *16S rRNA* was used as an internal control. **b**, **c** Relative expression of *CHH* in males and females, respectively. The relative expression level of *CHH* was significantly higher in the intermolt stage than in the premolt stage in males (**b**), whereas the relative expression level of *CHH* was significantly higher in the premolt stage in females (**c**). **d**, **e** Relative expression of *MIH* in males and females, respectively. The relative expression levels of *MIH* were not significantly different between the intermolt and premolt stages in both males and females. * and ** indicate significant differences at *p* ≤ 0.05 and *p* ≤ 0.01, respectively

RT-PCR and quantitative PCR analyses of *CHH* and *MIH* expression in the AnG were performed in males and females during intermolt and premolt stages. Significant differences in *CHH* expression were shown between the intermolt and premolt stages of both males and females (Fig. 11a, b, c), whereas there was no significant difference in the expression of *MIH* between the groups (Fig. 11a, d, e). The expression of *CHH* was decreased in males during the premolt stage (Fig. 11a, b), whereas it was increased in females (Fig. 11a, c).

**Fig. 12:**
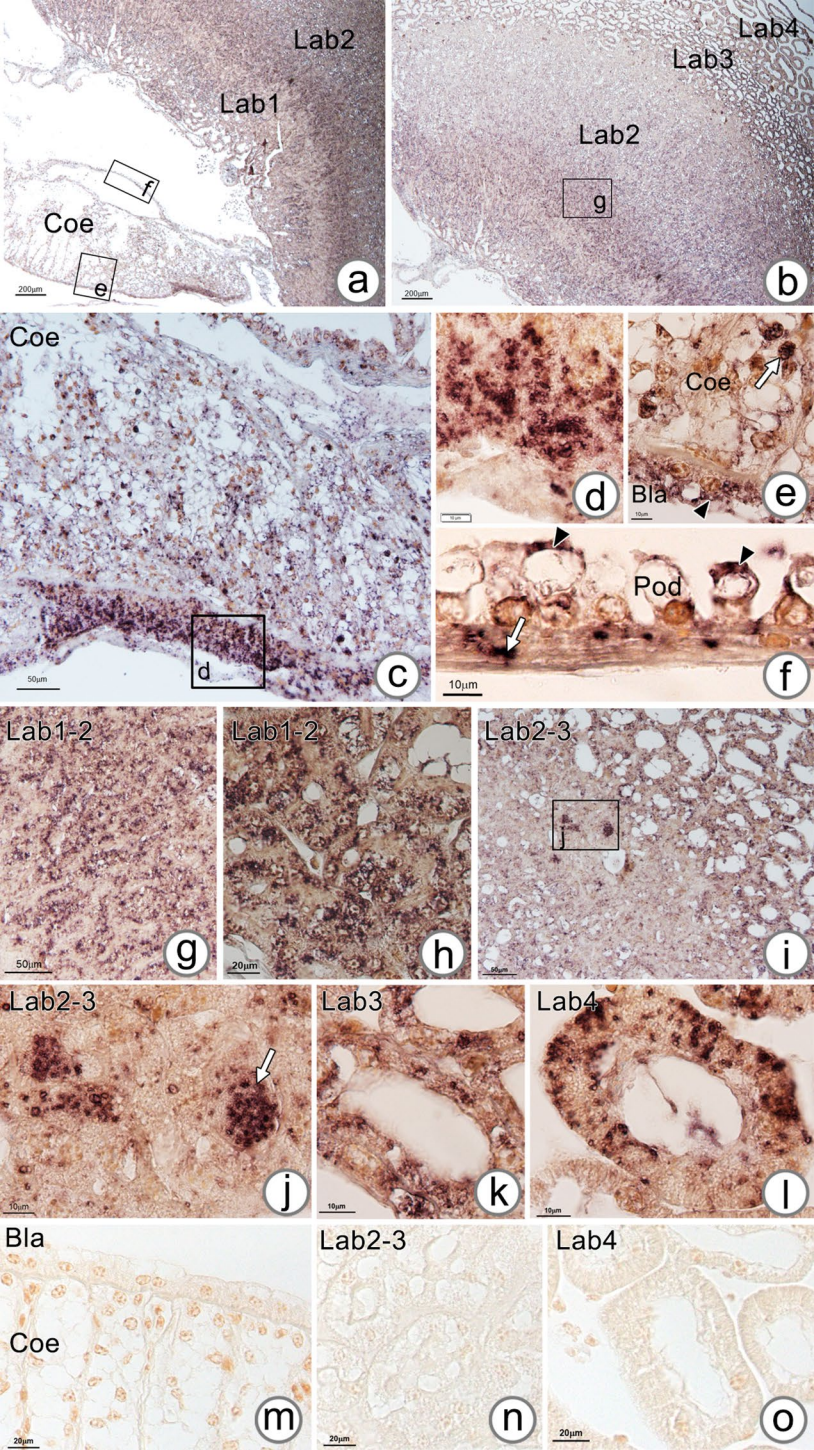
In situ hybridization of *CHH* in the AnG. **a**, **b** Overview image of the AnG showing *CHH*-positive signals in the Coe and Lab. (c-l) Positive signals of the *CHH* transcript are localized in the Coe and Bla epithelia. **d**–**f** Positive *CHH* transcript signals are observed in high columnar Bla epithelial cells (**d**), low cuboidal Bla epithelial cells (**e**, arrowhead), Coe stromal cells (**e**, white arrow), podocyte blebs (**f**, arrowhead), and podocyte processes associated with the vascular wall (**f**, white arrow). Moreover, *CHH*-positive signals were observed in the tubular epithelial cells of Lab1 and Lab2 (**g**, **h**) and some of the tubular epithelial cells of Lab2-3 (**i**) (**j**, arrow), Lab3 (**k**), and Lab4 (**l**). **m**–**o** Negative control sections in which an unlabeled probe was applied

In addition, the localization of *CHH* transcripts in AnG by in situ hybridization showed the expression of *CHH* in many regions of the AnG, including Bla (Fig. 12a) and Lab (Fig. 12b). At higher magnification, the *CHH-*positive signal was found in the epithelial cells of the Coe close to the vascular pole (Fig. 12c, d), Bla’s epithelial cells (Fig. 12e, black arrowhead), coelomosac stromal cells of Coe’s septa (Fig. 12e, white arrow), and the cytoplasmic protrusion (Fig. 12f, black arrowheads) and foot processes of podocytes at the basal lamina (Fig. 12f, white arrow). Moreover, the *CHH*-positive signals were predominantly localized in the epithelial cells of Labs1 and 2 (Fig. 12g, h), some tubular epithelial cells of Lab2 (Fig. 12i, j), Lab3 (Fig. 12k), and some regions of Lab4 (Fig. 12l). A negative control, where the unlabeled probe was used, showed only background staining (Fig. 12m–o).

## Discussion

### Structure of the antennal gland

In this study, we investigated the complex structural and cellular organization of the AnG in *M. rosenbergii*. Our research revealed the external and internal morphologies of the AnG by gross observation, SEM, and SR-XTM. In addition, the labyrinthine tubular types were classified based on H&E, PAS staining, and semithin sections. SR-XTM revealed the gross organization of the AnG by multiple serial sectioning data via computational tomographic analysis. The AnGs were connected to the uronephric duct, which opened at the nephopore at the base of the antenna. The bladder (Bla) was associated with the labyrinth (Lab), coiled ductules, and the end-sac or coelomosac (Coe). Moreover, tomography showed the great vessel, namely the antennary artery (McGaw and Reiber 2002), projecting into the Coe area. The location and complexity of the AnG of *M. rosenbergii* differ from the structure of AnG reported in the semiterrestrial crab, *O. stimpsoni* (Tsai and Lin 2014), in which Coe is present at the center of the Lab. However, the prawn’s AnG is quite similar to that of the lobster *H. gammarus* (Khodabandeh et al. 2005a, b, c), in which the Coe is close to the Lab and covered dorsally by the Bla.

### Cellular organization of the antennal gland

In previous studies, a few cell types of the AnG were mentioned based on their locations, including coelomosac cells, labyrinthine cells, end-labyrinthine cells, and bladder cells (Khodabandeh et al. 2005a, b, c; Tsai and Lin 2014). However, our results demonstrated a close association between the Coe and the outermost layer of Bla cells, which share the glycoprotein-enriched basal lamina (Fig. 6d). The Bla epithelial cells appear as a single layer of columnar to low cuboidal cells along the margin of the Coe toward the medial side. Only Pod has been reported as a major cell type in the Coe with a urine filtration role (Khodabandeh et al. 2005a, b, c). However, this study demonstrated that at least three cell types were present in Coe: Coe stromal cells, mesangial-like cells, and Pod. Coe stromal cells containing small vacuoles were found on the basal lamina associated with Bla cells (Fig. 6d), whereas mesangial-like cells were located within the septum (Fig. 6a, b). Pod was associated with the antennary artery and other smaller vessels in the Coe septa (Fig. 6e). Sizeable vacuoles and many Pod cytoplasmic blebs, previously mentioned as lysosomes (Ueno and Inoue 1996), were observed (Fig. 6c and e). The shape of crustacean Pods has been reported to resemble a spider or octopus (Ueno and Inoue 1996; Khodabandeh et al. 2005a, b). However, the appearance of Pod in our report might be an early phase of metabolic waste absorption. In *Neocaridina denticulata*, trypan blue was injected and observed in small vesicles before secretion into the urinary space via apical protrusion (Ueno et al. 1997).

Mesangial cells in vertebrate nephrons play an essential role in supporting the glomerulus, producing many vascular mediators, and handling macromolecules (Schlöndorff 1996). Generally, mesangial cells have a mesangial matrix positively stained by PAS (Kierszenbaum and Tres 2012; Jeong 2020). However, the presence of mesangial cells in invertebrates remains unknown (Ichimura and Sakai 2018). Our study proposed that the cells located within the Coe septum might be mesangial cells because of their intense PAS staining. Recently, studies in mice and humans demonstrated that *Pdgfrb* is specifically expressed in intraglomerular mesangial cells (He et al. 2021). Therefore, further investigation is needed to examine whether it can be used as a marker of mesangial cells in invertebrate kidneys.

Previous reports have demonstrated that the Lab consists of Lab cells forming a tubule, but the detailed cellular structures are not well established (Al-Mohsen 2009; Khodabandeh et al. 2005a; Tsai and Lin 2014). In *M. rosenbergii*, the ultrastructure of Lab tubular epithelial cells has been demonstrated based on TEM (Al-Mohsen 2009). However, our study revealed that the Lab tubules presented a heterogeneous group of cells based on the location of tubular zones. Based on H&E staining, Lab tubules could be divided into four zones. Lab1 cells are irregular in shape, have a high brush border at the apical surface, high aggregation of nuclei, and dominant mitochondria in the cells. These cellular features reflect the reabsorption function using the energy from the filtrated urine in the urinary Coe space, possibly by ATP-dependent pump (Khodabandeh et al. 2005c). Lab2 could be distinguished into two zones: the proximal part, located close to Lab1, and the distal part, located close to Lab3. Moreover, those zones could be classified as tubular structures in four categories based on cellular characteristics: type 1, most tubular epithelial cells carry large vacuoles; type 2, tubular epithelial cells have small vesicles and enriched mitochondria; type 3, two types of tubular epithelial cells, dark and pale cells; and type 4, tubular epithelial cells carry many small vacuoles. However, the function of each tubular type has not been investigated and requires further study. Lab3 was pale stained by H&E and toluidine blue staining, whereas Lab4 was categorized into two distinguished tubules. The function of the Lab3 tubule remains unclear; however, based on the histology of its cells, which carried a brush border at the apical surface and mitochondria in the basal part, this section might be involved in an absorptive function. Histologically, tubule type 2 of Lab4 appears like the distal convoluted tubule in mammals with extensive basal laminar infoldings with numerous associated mitochondria and scant brush borders on the apical part (Bulger 1986; Kierszenbaum and Tres 2012). However, the function of type 1 of Lab4 remains unclear because of its unique characteristics.

### Expression of vertebrate kidney-associated homolog in the antennal gland

In this study, a functional reflection of the AnG as a kidney equivalence was demonstrated by the expressions of *AQP*, *SLC22*, *nephrin-like*, and *uromodulin-like* genes. These four genes have been identified in the AnG transcriptome analysis by Bose et al. (2017). In situ hybridization showed *AQP* and *SLC22* transcripts expressed in the Bla epithelial cells and some parts of Lab tubules, especially in some regions of Lab1, Lab2–3, and Lab4. The *SLC22* transcript was predominantly expressed in the interjunction of Lab tubules 2–3. The expression level of AQP was relatively similar between males and females and was predominantly expressed during the premolt stage as compared to the intermolt stage. This occurrence can be attributed to the preparation for molting, which increases osmotic permeability. This has been studied in the muscles of *Palaemonetes argentinus* by Foguesatto et al. in 2017. The relative expression of *SLC22* was slightly lower in the intermolt females than in the males but was not significantly different. The putative roles of *SLC-22* in terms of kidney function in crustaceans remain unclear. However, the existence of the SLC superfamily has recently been reported in *D. melanogaster* and showed conservation with human SLCs (Ceder and Fredriksson 2022). SLC22 belongs to a member of the organic ion transporter family, playing an essential role as a determinant mediator of the absorption and disposition of many prescription drugs in humans (Yee and Giacomini  2022). Nephrin and uromodulin are proteins that typically perform their function in the kidneys of vertebrates. Uromodulin is produced from the renal epithelial cells lining the thick ascending limb of Henle’s loop. This molecule is involved in the endoplasmic reticulum’s homeostasis and the protein’s unfolding response (Schaeffer et al. 2017). Nephrin is an integral protein associated with the slit diaphragm of podocytes and functions to stabilize the slit diaphragm in the vertebrate kidney (Li et al. 2013). In this study, we reported the expression of nephrin-like and uromodulin-like genes in the *M. rosenbergii* AnG for the first time. Although the functions of these genes were not investigated in this species, they might be involved in kidney-related functions according to the conservation of molecular function during animal evolution. The presence of the nephrin gene in the fruit fly *D. melanogaster* (Weavers et al. 2009) indicates that these molecules might also be conserved in crustaceans.

### Expression of neurohormones in the antennal gland

Neurohormones are generally found in the central nervous system (Dircksen et al. 1988; Chen et al. 2020). However, several studies have demonstrated the presence of some neurohormones in crustacean AnG (Chen et al. 2004; Nguyen et al. 2016; Zhang et al. 2020). In this study, we found some contigs and unigenes of two neurohormones, *CHH* and *MIH*, in the transcriptome data of *M. rosenbergii’s* AnG, suggesting their roles in this organ. The expression of *MIH* has been previously found in the tegumental gland, which is a non-neural tissue of the shrimp *P. monodon *(Namvongsakool et al. 2015). In fact, the expression of *CHH* has previously been reported in the AnG of *M. rosenbergii* (Chen et al. 2004). However, the cellular localization of the gene transcripts has never been demonstrated. This study was the first to report the cellular production of *CHH* in the AnG of *M. rosenbergii* by in situ hybridization. The results showed broad expression of *CHH* in several areas of the AnG, including the Bla, Coe, and podocyte epithelium and tubular epithelial cells of Lab1, interjunction of Lab2–3, and Lab4. Moreover, RT-PCR and qPCR analyses revealed different expression levels of *CHH* in males and females during the molting stages. The relative expression of *CHH* was high in males during the intermolt stage and in females during the premolt stage. In contrast, the relative expression of *MIH* was not significantly different in the AnG of males and females during different molt stages. Neurohormones produced from the eyestalk, brain, and thoracic ganglion have been reported to affect salt and water transportation in the AnG (Kamemoto 1976). A study on the Christmas Island blue crab, *Discoplax celeste*, revealed that the CHH peptide can increase AnG urine production (Turner et al. 2013; Turner 2014). Moreover, CHH plays a role in stimulating Na+ transport in the gill epithelial cells of the Christmas Island blue crab *D. celeste* (Turner et al. 2013). In addition, CHH seems to perform an essential function relevant to the molting stages. In the crab *Carcinus meanus*, CHH is involved in the molting cycle (Chung et al. 1999). Gut-derived CHH is highly produced and released into the hemolymph during late premolt and ecdysis, suggesting that CHH promotes water and ion uptake, resulting in body swelling during ecdysis (Chung et al. 1999; Chen et al. 2020). This study demonstrated the presence of neuropeptides, at least *CHH* and *MIH*, in the AnG of *M. rosenbergii*. Further investigation is required to understand the roles of neuropeptides in the AnG.

In conclusion, this study reported the detailed organization of the AnG in *M. rosenbergii* by SR-XTM and classical SEM. We also described the histological classification of the Lab and the cellular component of the Coe and Bla. In addition, we demonstrated that some vertebrate kidney-associated homolog genes (*AQP*, *SLC-22*, *nephrin*, and *uromodulin*) and neuropeptides (*CHH* and *MIH*) were expressed and localized in *M. rosenbergii* AnG. The finding of neuropeptide expressions in the AnG implied that the AnG may have a role in hormone production and that these hormones may be involved in the AnG’s functions. Moreover, male and female prawns exhibited different levels of some gene expressions (*AQP*, *SLC-22*, *CHH*) during different molt stages, suggesting a crucial role relevant to the molting stages. Nevertheless, more research is required to determine the function of these genes, especially during ecdysis.

### Supplementary Information

Below is the link to the electronic supplementary material.

Supplementary file1 (WMV 5505 KB)

Supplementary file2 (WMV 6797 KB)

Supplementary file3 (DOCX 14 KB)

## Data Availability

Data supporting this study are openly available from the AnG transcriptome database (SRA number: PRJNA381087), GenBank accession number AF219382.1, GenBank accession number KC990939.1, and included within the supplementary data.
