# Supplementary material for: Anatomical and molecular insights into the antennal gland of the giant freshwater prawn Macrobrachium rosenbergii
Source: Cell Tissue Res. 2024 Jun 15;397(2):125–46. doi: 10.1007/s00441-024-03898-3 (PMC11291661; doi:10.1007/s00441-024-03898-3)
Supplement: Supplementary file 3 — Supplementary file3 (DOCX 14 KB) [file 441_2024_3898_MOESM3_ESM.docx]

**Supplement 3_** nucleotide sequences selected from the Antennal gland Transcriptome_*Macrobrachium rosenbergii*_Kruangkum et al.,2023

>Unigene20598_nephrin

GAGAGATGGTGGGGACTGTGGGACCCCTTCAGGAGAACCAGCTGACGGAACTTACCTGTAGGAGTGTTGGGGGGCGGCCTTCTCCTACCCTAACCTGGTGGAGTGAGGGCAGAGAGCTGCCTCTTCTCTACGCTAATTCCTCTTTGGATCCTGTGACAGGAACGAGTGTGGTCGAAGCCACCTTGTCAGTGACGGCCAAAAGAGAACTCCAGGGTGGATCTTTGACGTGTTACGCCCTCACGCCCAGTCACCTCAACGCCTCCGATGCTGCCACAGTTCCCCCTCGGAGTGCCTCAGTGTCACTCAACATCACACTCTCCCCAGTGGAAGTGCGGATACTGGAACCAAACCCTATCGTAGCCACTTCAGGGACCACCATCAACATCGTCTGTCGCGCCCTTGGGTCGCACCCTCCTGCAGATCTCTCCTGGTGGAGGGGCATAAGGTCCCTGGAGCCCCATGTTACTCACGCCATACAAGACGGTGGTAACATCACAACAGCCACTCTGACCGTCTCCGTGACGGGCGAGGATGACGGAGCTACAGTCACCTGCACAGGTGCGAATCCTGCTCTCTCCCGCGAGGAGCCTCTGTCAGACAGGAGAAAGCTCATTGTCTACTGTGAGTTCAAGAGGTAATTTGCTTGCGCCTTCGCTC

>Unigene16991_solute carrier family 22 member 8

CACTTGGCTCAGCTACGATCGCTTCACGAGTAGGGTCAATGACGTCTCCATTTATCACAGAATTGCTGGGTCCACTTTATCCAAGGGCACCATCTATCTTATTTGGCATATCAGCATTTGTAGCGGGAATAGCTACAATTCCTCTGCATGAAACTTTAGGCAGACCTCTACCTGATACTATTACAGACCTGGAGAAACCTCAGATACAGGAACCTGAAGAACCAGATGAAGAAACAGAGATGGCTAAACTCCGGGCGTAGTTAGTCTACAAATCTGTGATGAAACTTTTCTATTCTCTCAAGTTCCCTCAACCACAGTCGTGCTGTTCTACCGAACAAGCTCAGCGCATGATCTAC

>CL1148.Contig1_Uromodulin

GCTAGAGTGGTTGTTTACGACTGTTCTCGAGATGTGCTATATCAAACTGAACGTGTTCTTGGCCGCTGTAGCTCTGTCTTCTTTGTGTAAAGCTCAGATGATTTCTCAGCAGGAACAAATCACCGTACTACTCACAAACCTTAATGGAAAATTAGAAGGTCTACAACAAGCTCTGGAATCGATTGAGCATACACAAAAGTACATTGAGCAAAGTATTCAGAATCAACAATCGCAACTATCTTCAATAGAAGAGTCGCAGCTGGCGATGGGAAAAGAAGTGCAAGGGTTGCAGGATTTCATATCGTCTCAGAAGTCCCCTTTATGTAAAGACGACTTCAGTGATTTTAAGAGTTTACTTACAAAAGATGAGTGTATGGACGGAACCCACAACTGTAGTCGGTACGCAGAGTGCAAGGATACGATGCTTAGCTTCGCCTGTTCATGTCTACCTGGTTACTCCGGTGATGGGTTCGAATGTTCAGATATTAATGAATGCACAGATGCGGACATTTGTGGAGATCGCACAAGTTGCAAAAATGTGGAAGGATCCTACAAATGCGAGTGTTCAAATGGCTACGAAATGAAAAACGGCAGGTGCCAAGATGTCGACGAATGCACGCGAGAAGGCTCACACGAGTGCCAGGCTCCAGCCACATGCGTCAACTCTGAAGGCGGCTACGAGTGCCAGTGTCTGCCACCTTACAAAGGAGATCCGTTGAATTGCGGGATGGAATGTTTGCCCCAGTCTCTCTACATAAGAGGCCTGGGTTGCATCACGCCTATGCTCACACGGCTCCCCTGGGAAGAGGCAAAGCGAGTATGTGAAGGATCCGGCGGCAGATTGCTGGAAAACATCGAGAACTTGCATTTTGAAGCCATCAGTCAACATTTCAAGCCTCTCATGAGCAATCGTCCCTTTCCATGGATTGGTTTCAAGAACAAGGCCTGGGTCTCCACTGGGCGTGCCGTTTCGAAAGAGGTCGTGGCGAGGCAGGAGGACGATCTGGAGGGAGACTGTGGTAATATCGATCTGAGAGAGACCCAGCTGGGACTGTGGGACGCGAGCTGCTTCGACGAGGAATATGCGCTGTGTCAGAAAGTCTAACATTTAATGATCTGATTCCCACAGAGAAAAGTCACTCACAAAGTGCCTTGCCTGTTGCACTGTTATCTAAACGCGCTTATAAGTTCTTTCCCCAAATGCCTTGCTTTCTGTCTTCTAATATACCTTCCCTCCCTCTCGTTTCTTCCTGCCTTGCTGCCTAACTTCTACGTCATCTTGCTCCAAATTCCAGCTTTTGTTTTCGAAAATTCAGCATTAGCCGCAACAGTATGAACATTTCTAATAAAATGAAG

>Unigene28443_AQ

ATGGAGGTGCCCAAGTACTTGATTCCTCTCTTCGTCGGCTTCACCGTCGTGAACATCGGCATCTGCTTCGGCTTCAACTGCGGCTACGCCATCAACCCCGCCAGGGACCTGGCACCCAGACTCTTCACCCTCATTGCCGGATGGGGAGGCGATACGTTCAAAGCAAGCACCTTCGAGGGAATTGTCTGGTGGTGGGTGCCCATTGTTGGCCCACACATCGGAGCCGTCTTGGGCGTGGCGATATACCTGGCGCTGATCGAACTCCACCACCCGGAGGCACAGGAGTTCTCCCTTCCTCAGATCCACCAGTCACAAGATTCAGTGGCCACAGCCTCG
